# Heat Shock Protein Overexpression-Mediated Periodontal Ligament Regeneration: A Fundamental Approach to Generate a Potential Biomaterial

**DOI:** 10.3390/ma15030809

**Published:** 2022-01-21

**Authors:** Rina Muraoka, Keisuke Nakano, Toshiyuki Kawakami

**Affiliations:** 1 Department of Orthodontics, Matsumoto Dental University School of Dentistry and Hospital, 1780 Hirooka-Gobara, Shiojiri 399-0781, Japan; 2 Department of Oral Pathology and Medicine, Graduate School of Medicine, Dentistry and Pharmaceutical Sciences, Okayama University, 2-5-1 Shikata-cho, Okayama 700-8525, Japan; keisuke1@okayama-u.ac.jp; 3 Department of Oral Pathology, School of Dentistry, Aichi Gakuin University, 1-100 Kusumoto-cho, Chikusa-ku, Nagoya 464-8650, Japan; kawakami.emailbox@gmail.com

**Keywords:** biomaterials, fibroblasts, heat shock protein (HSP), homeostasis, mechanical load, periodontal ligament (PDL) tissue, regeneration, remodeling

## Abstract

The periodontal ligament (PDL) is a cell-rich fibrous connective tissue supporting the tooth roots. The tissue helps to maintain homeostasis and exhibits regenerative and repairing ability, which is mediated by the heat shock protein (HSP). Here, we experimentally created PDL tissue with notable ability to regenerate hard tissue and evaluated it as a potential biomaterial. We immunohistochemically examined the mechanical load-induced HSP overexpression in mouse PDL. Following mechanical load application and release, HSP70 localization in the PDL was altered immediately, suggesting that the HSP70 function may differ with the timing of its expression in PDL. HSP70 expressed in the cytoplasm and nucleus of fibroblasts in PDL on the tension side not only participated in periodontium repair, but also functioned as a molecular chaperone during protein expression involved in osteogenesis to restructure injured tissue. This study highlights the potential of artificially created highly functional PDL tissues as biomaterials.

## 1. Introduction

Understanding the dynamics of various factors expressed in the periodontium is extremely important for providing a biological basis for treatment approaches. Mechanical load induces the expression of various protein molecules and causes periodontium remodeling. Orthodontic tooth movement results from periodontal ligament (PDL) remodeling, which is caused by bone resorption on the side subjected to constant pressure and bone formation on the side subjected to tension caused by mechanical load [1,2]. The PDL is a cell-rich fibrous connective tissue supporting the tooth roots, which is involved in maintaining tissue homeostasis and displays notable regenerative and repairing ability.

The principal constituent of the extracellular PDL matrix is collagen, which alleviates mechanical load, such as occlusal force, orthodontic force, and mechanical corrective force. The collagen fibers in the PDL are deformed after the application of a mechanical load, and return to their original width once the load is released. Compared with the bone marrow used for live stem cell transplantation, PDL is clinically easier to collect and less burdensome to patients. Biologically, it is a stronger tissue than cultured cells and can be transplanted to various sites. To maintain homeostasis, the periodontium expresses various proteins involved in periodontal remodeling in response to different stimuli, such as mechanical load and inflammation [3,4,5,6,7]. Experimental tooth movement causes PDL remodeling, which temporarily alters the distribution and number of cells present in the PDL tissue [8,9]. The periodontal tissue is composed of cranial neural crest (NCC) derived ectomesenchyme. The dental papilla and dental follicle are formed by ectodermal mesenchymal cells, which give rise to the periodontal tissues, dentin, and dental pulp. Dental follicle stem cells (DFSCs) derived from ectodermal mesenchymal cells function as dental mesenchymal stem cells (MSCs) that proliferate to form PDL fibers. In contrast, DFSCs also differentiate into osteoblasts, forming alveolar bones. Moreover, some studies on embryological involvement have analyzed the regeneration of periodontal tissue lineage cells [8]. However, little is known about the cellular proteins contributing to these regeneration and recovery mechanisms. Based on its properties, we speculated that PDL tissue may be used as a biomaterial for tissue repair.

We have previously reported that heat shock protein (HSP) 70, which participates in the recovery response to cellular injury in various tissues, is expressed in the mouse periodontium at a very early stage following cellular injury to maintain homeostasis [6]. HSPs are the main proteins expressed in various organs and tissues after exposure to cell injury stimulus, such as mechanical load [10,11,12]. HSPs are essential for various cellular activities, such as cellular differentiation, proliferation, survival, and maintenance. The HSP family is involved in maintaining homeostasis and PDL regeneration and HSP70 is constitutively expressed in PDL to maintain its physiological function by protecting it against various cellular stresses [13,14]. HSPs are polypeptides with different functions, range from several kDa to several hundred kDa, and are classified based on their molecular weights. High-molecular-weight HSPs, such as HSP70 and HSP90, act as molecular chaperones that function in protein maturation by temporarily binding to immature proteins to mediate polypeptide folding and association. Most HSPs are molecular chaperones [15] that suppress protein denaturation and repair denatured proteins, and exhibit anti-apoptotic functions to prevent cell death. HSP70, investigated in this study, is an ATP-dependent HSP that facilitates the correct folding, transport, and degradation of new proteins and suppresses their denaturation [16,17,18]. In other words, HSP70 also processes and repairs denatured proteins, disposing denatured proteins that cannot be repaired. HSP70 is a major protein involved in cellular quality control processes.

Although the periodontal ligament is a highly regenerative tissue, it actively acts in tissue repair when it is subjected to moderate stress and injurious stimuli, as seen in orthodontic tooth movement. HSPs are representative stress proteins that are expressed under conditions of cellular damage, and are involved not only in repairing damaged cellular functions, but also in inducing cell differentiation. Among them, HSP70 has been reported to induce osteoblast differentiation. The periodontal ligament, which overexpresses HSPs, may function actively as a biomaterial with high regenerative capacity. In particular, the expression of HSP70 is desirable for hard tissue regeneration. Therefore, we investigated when and where HSP70 was expressed in the periodontal ligament after mechanical loading.

This study consists of basic research to confirm the expression and change of HSP and the expression of tissue repair related factors in order to investigate the possibility of application of PDL as a biomaterial with special functions.

## 2. Materials and Methods

### 2.1. Experimental Animals

Forty-five 8-week-old male ddY mice (body mass 35 ± 5 g) were used as the experimental animals. The mice were kept in an environment controlled by air conditioning (24 ± 1 °C) and reared in metal cages. The mice were fed freely with water and solid diet during breeding. All animals used in this study were cared for in accordance with the experimental animal guidelines of the Matsumoto Dental University Animal Study Ethics Committee.

### 2.2. Experimental Methods

**Figure A1:**
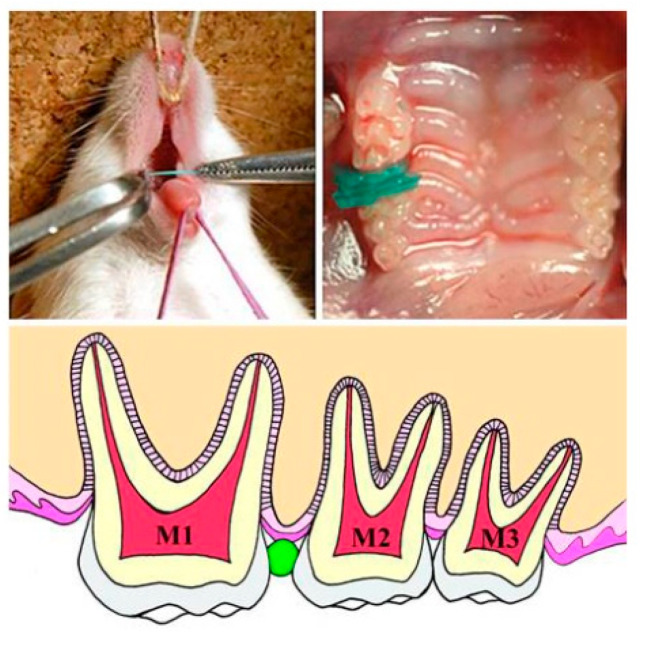
Schematic explaining the insertion. The upper jaw was immobilized by fixing a kite string and the lower jaw was immobilized by fixing a rubber band. Copyright, 2010, Rina Muraoka.

The Waldo method [19] was used to insert a separator to provide the mechanical load to the maxillary molar periodontium of the mouse for 3 h (Figure A1 in Appendix A). Then, the separator was removed, and the periodontium was collected from the maxillary molar region of five mice immediately or after 20 min, 1 h, 3 h, 9 h, 24 h, 3 d, and 1 week (3 h + 0 min, 3 h + 20 min, 3 h + 1 h, 3 h + 3 h, 3 h + 9 h, 3 h + 24 h, 3 h + 3 d, and 3 h + 1 w, respectively; Table 1). After removing the mechanical load, the mice were allowed to consume water and food pellets freely until the end of the experiment. We examined the mechanical load caused by the insertion of the separator and conducted this experiment assuming that after this force was removed, there would be no pressure and tension caused by the viscous behavior of the PDL in the periodontal ligament.

The periodontium from the left maxillary molar region (the opposite, untreated side) of the same mouse was used as control. The distobuccal root of the first maxillary molar for both the experimental and control groups was observed. The study was conducted according to the guidelines of the Animal experiments of Matsumoto Dental University and approved by the Matsumoto Dental University Animal Study Ethics Committee (Approval No.: 179-10; date of approval: 2010).

### 2.3. Immunohistochemical Investigation of HSP70

After removing the maxilla with the maxillary molar periodontium from the mouse, they were quickly submerged in a fixing solution (4% paraformaldehyde 0.05M phosphoric acid buffer) for 24 h, and then decalcified (10% EDTA demineralizing solution) for 3 weeks. Next, the samples were embedded in paraffin and 5-μm-thick serial horizontal sections were produced from the root PDL before immunohistochemical investigation.

A DAKO EnVision + Kit-K4006 (Dako; Glostrup, Denmark) was used for the immunohistochemical analysis. Anti-mouse HSP70 polyclonal antibodies (HSP70 (K-20): sc-1060-R; Santa Cruz Biotechnology, Inc.; Santa Cruz, CA, USA; dilution ratio, 1:1000) were used as primary antibodies for immunostaining and hematoxylin was used as the counterstain. The positive reaction of the primary antibody was confirmed by coloration with 3,3’-Diaminobenzidine (DAB). Additionally, the negative controls were stained using the same procedure, but without using primary antibodies. 

## 3. Results

### 3.1. Immunohistochemical Investigation

#### 3.1.1. Control Group

**Figure 1:**
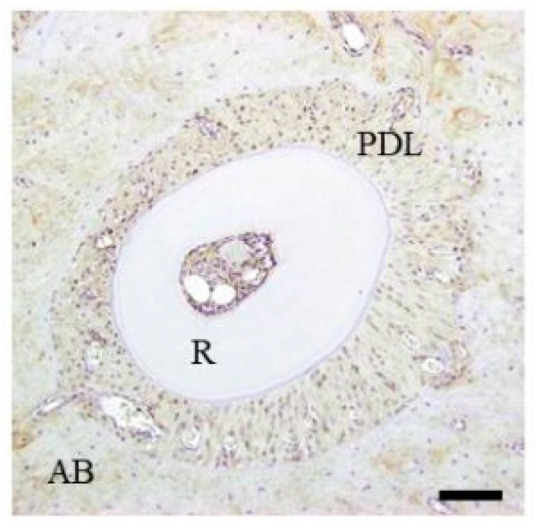
Immunohistochemical staining profile of HSP70 in control specimens. AB, alveolar bone; PDL, periodontal ligament; R, tooth (dental root). The inset bar indicates 50 μm.

In the PDL tissue from mice in the control group (murine upper left first molar distobuccal root), HSP70 expression was detected in the cytoplasm of PDL fibroblast uniformly over the entire PDL. However, these nuclei were not stained by DAB on either the tension or pressure sides of the control group (Figure 1).

#### 3.1.2. Experimental Group

**Figure 2:**
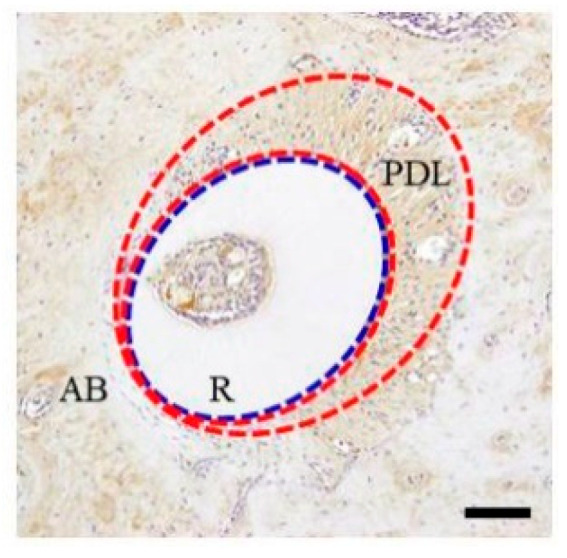
Immunohistochemical staining profile of HSP70 in 3 h + 0 min group specimens. AB, alveolar bone; PDL, periodontal ligament; R, tooth (dental root). The inset bar indicates 50 μm. Blue dotted line; Boundary between periodontal ligament and alveolar bone. Red dotted line, HSP-positive area.

After releasing the mechanical load for 3 h, the maxillary molar periodontium was collected sequentially for up to one week. The change in HSP70 expression in the mouse periodontium from the investigated region was observed immunohistochemically. After releasing the mechanical load, the tooth roots were relatively displaced in the anterior direction and the PDL space was narrowed on the pressure side immediately after separator removal (3 h + 0 min). The PDL space was enlarged by the same amount on the tension side and the periodontal fibers in the area were considerably extended. In some PDLs, the periodontal fibers were sparse and contained gaps in some places with torn periodontal fibers. HSP70 localization was observed in PDLs on both tension and pressure sides. Specifically, HSP70 expression was greater on the tension side than that in the control group (Figure 2).

HSP70 expression changed time-dependently after releasing the mechanical load. Twenty minutes after releasing the mechanical load, HSP70 expression in the tension side was similar to that immediately after stress release. The teeth roots moved slightly backward to their original position in the alveolar bone and HSP70 expression occurred during PDL surface recovery on the pressure side. HSP70 expression in the pressure side increased over time and increased in both the pressure and tension sides 3 h after release (3 h + 3 h). HSP70 expression was similar in both the pressure and tension sides 24 h after release (3 h + 24 h). However, it increased in the pressure side 3 days after release (3 h + 3 d). One week after release (3 h + 1 w), HSP70 expression decreased and was similar in both the pressure and tension sides.

**Figure 3:**
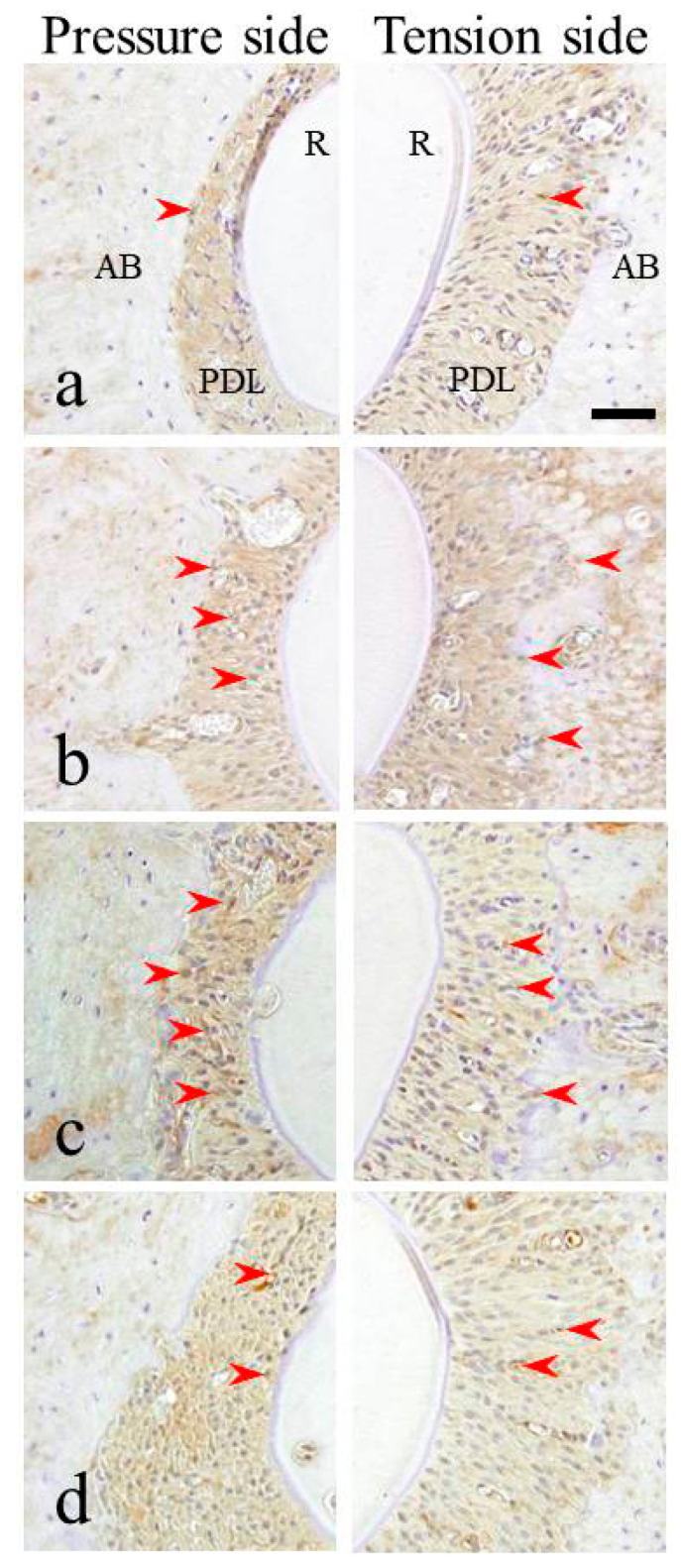
Immunohistochemical results at horizontal sectioning of the root portion. (**a**) 3 h + 20 min group, (**b**) 3 h + 24 h group, (**c**) 3 h + 3 d group, (**d**) 3 h + 1 w group. AB, alveolar bone; PDL, periodontal ligament; R, tooth (dental root). The inset bar indicates 50 μm. Arrowheads indicate periodontal ligament cells with relatively strong positive reactions.

In summary, HSP70 expression in the PDL of the tension side was greater than that in the pressure side at the very early stages after mechanical load release. However, HSP70 expression gradually increased in the PDL of the pressure side and was greater than that in the tension side 3 days after release (3 h + 3 d). One week after release (3 h + 1 w), strong HSP70 expression was maintained in all areas of the PDLs (Figure 3). The immunohistochemical results are summarized in Table 2.

After releasing the mechanical load (3 h + 0 min), the nucleus was not stained by DAB in either the tension or the pressure sides (Figure 2). After releasing the mechanical load exerted for 3 h, time-dependent expression changes were observed in the nucleus. First, 20 min after releasing the mechanical load, DAB-stained nuclei were observed (Figure 3). DAB-positive nuclei increased over time. HSP70 expression was similar in both the pressure and tension sides after 24 h (3 h + 24 h). Approximately 50% nuclei were DAB positive. Three days and 1 week after release (3 h + 3 d and 3 h + 1 w, respectively), HSP70 expression decreased and was similar to that in the control group.

## 4. Discussion

Experimental tooth movement causes PDL remodeling, which temporarily alters the distribution and number of cells present in PDL tissue [20,21]. We have previously reported that HSP70, which is thought to participate in the recovery response to cellular injury in various tissues, is expressed in the mouse periodontium at a very early stage after cellular injury of the periodontium, to maintain homeostasis [6]. HSP70 is constitutively expressed in PDL to maintain its physiological function by protecting against various cellular stresses. However, HSP70 function depends on the timing of its expression and its intracellular distribution. The cell characteristics and associations that are expressed in the nucleus and the cytoplasm are different. The difference in the intracellular HSP70 distribution is due to the difference in HSP function.

Intracytoplasmic positivity of HSP70 is associated with cell differentiation. This is inferred from the cytoplasmic findings of fibroblasts and osteoblasts during maturation and differentiation [4,5,6,7,8,9]. Nuclear HSP70 action requires nuclear HSP40 expression [3]. Nuclear positivity of HSP70 is involved in recovery from cytotoxic damage.

The mechanism of tooth movement during orthodontic treatment has been widely examined [1,2]. However, few studies have examined the cellular responses related to recovery from injury-induced mechanical load to PDL fibroblasts during orthodontic treatment. We have previously identified various proteins expressed in the mouse periodontium after mechanical load mimicking orthodontic treatment [3,4,5,6,7,8,9]. Here, we focused on HSP70, a ubiquitous and highly conserved protein that maintains cellular homeostasis and is expressed after various types of cellular injury.

We investigated changes in HSP70 expression in PDL fibroblasts. HSP70 is expressed on the osteogenic side under tension, likely because of its role in protein repair. HSP70 localization on the pressure side, which undergoes bone resorption, participated in denatured protein processing [6]. 

Since HSPs are thought to be strongly expressed during the tissue repair process, we examined the expression of HSP70 after stimulation of the periodontal ligament with a strong load. This method is useful for the application of periodontal ligament cells with the strongest HSP expression as biomaterials.

We investigated the periodontium recovery at different times after releasing the mechanical load on the mouse periodontium until after 1 week.

Immunohistochemical analysis showed that a homogenous and low HSP70 peptide activity was observed in the cytoplasm of PDL fibroblasts across the PDL region in the mouse periodontium of the control group.

Over the course of a typical day, the teeth are subjected to mechanical load due to biting many times. In such circumstances, the periodontium, which supports the teeth, maintains its physiological function. The present findings regarding the periodontium of the control group agree with those of previous reports [3,4,6], demonstrating that other HSPs, such as HSP27 and HSP47, are expressed under load-free conditions and maintain the periodontium function. Similar to these proteins, HSP70 is expressed under load-free conditions, most likely to maintain the physiological function of the periodontium and PDL homeostasis [6]. We believe that physiological occlusal force is one of the reasons why HSP expression is maintained for a long time. We have previously reported that HSP70, expressed on the pressure side, which receives considerable cellular injury, participates in osteoclast differentiation on the pressure side [6]. HSP70 may function in the repair and suppression of denaturation of new proteins and process denatured proteins that cannot be repaired [22,23,24].

In the very early stages after the release of the mechanical load, HSP70 expression level increased and decreased in the PDL on the tension and pressure sides, respectively. However, as the gap around the pressure PDL recovered to its original size with time, HSP70 expression increased in the PDL of the pressure side, and at 3 days after release (3 h + 3 days) was greater than that of the PDL on the side under tension. These responses are thought to be induced by the mechanical load on the PDL.

The expression of HSP70 was higher in the pressure side than in the tension side 3 days after the removal of load. It is possible that the cell injury was stronger in the compression side than in the traction side. As a result, the state of cell injury was different in the compression side compared to the traction side, and the expression of HSP70 was also different. In other words, the periodontal ligament on the compression side seems to be a promising material for tissue regeneration, but this point needs to be further investigated.

HSP70 contributes to the expression of Runx2 and Osterix [25]. These proteins, such as Runx2 and Osterix, are essential factors for bone differentiation. In other words, HSP70 is involved in the expression of proteins essential for bone formation by osteoblasts progressing. We considered the effectiveness of HSP as a biomaterial: a portion of the tissue overexpressing HSP70 is harvested and transplanted to a site requiring tissue repair. It will function as HSP70-positive PDL cells, at least during the period of expression of HSP70. HSP70 is physiologically expressed in the living body [3,4,6], and there is little risk when it is used as a biomaterial. HSP overexpression in the PDL is induced by a mechanical load to create a PDL tissue with a high ability to regenerate hard tissue. This study suggests the possibility of using these artificially added highly functional PDL tissues as biomaterials.

## 5. Conclusions

The function of HSP70 varies depending on the timing of its expression and its subcellular distribution. HSP70 expressed in the cytoplasm and nucleus of fibroblasts in the tensorial PDL is not only involved in periodontal ligament repair, but also functions as a molecular chaperone during the expression of proteins involved in bone formation, which may contribute to the reconstruction of damaged tissues.

HSP70 overexpression is induced in the PDL by a mechanical loaded model to create a PDL tissue with a high ability to regenerate hard tissue. This study suggests the possibility of using these artificially added highly functional PDL tissues as biomaterials.

## Figures and Tables

**Table 1:** Experimental periods and number of specimens.

Periods	Cont.	3 h + 0 min	3 h + 20 min	3 h + 1 h	3 h + 3 h	3 h + 9 h	3 h + 24 h	3 h + 3 d	3 h + 1 w
Numbers	5	5	5	5	5	5	5	5	5

**Table 2:** Immunohistochemical analysis of HSP70 expression strength.

Side	Time Course							
	3 h + 0 min	3 h + 20 min	3 h + 1 h	3 h + 3 h	3 h + 9 h	3 h + 24 h	3 h + 3 d	3 h + 1 w
Tension	+	+	++	+++	+++	+++	+++	++
Pressure	−/+	+	++	+++	+++	+++	++++	++

Immunopositivity: −, negative; −/+, weak; +, mild; ++, moderate; +++, strong; ++++, very strong.

**Figure A2:**
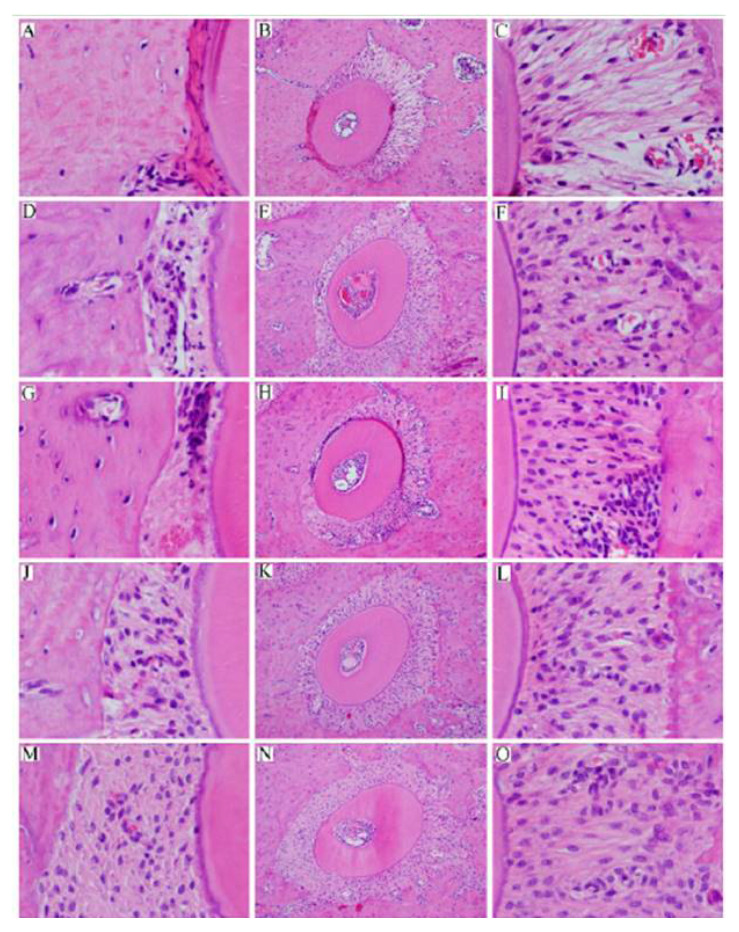
(**A**) Tooth root is close to the bone, PDL bundles are compressed and the PDL space show narrow, with degenerative cell changes (3 h + 0 min). (**B**) Tooth root moved towards the mesial, there is narrowing of the PDL space in the tension side, there is widening of the PDL space in the pressure side (3 h + 0 min). (**C**) Breaking of the PDL bundles occur and a space occurred in between bundles. The number of PDL fibroblasts is significantly decreased; there is dilation of hyperemic capillaries (3 h + 0 min). (**D**) Increase in the number of cells that are haphazardly arranged. Hyaline degeneration can be seen (3 h + 1 h). (**E**) Tooth root moved towards the distal PDL bundles in the tension side and stretched. (3 h + 1 h). (**F**) PDL bundles are stretched. Increase in the number of cells; the cells remain haphazardly arranged. There is still dilation and congestion of capillaries (3 h + 1 h). (**G**) Evident hyaline degeneration can be observed and there are only few cells (3 h + 9 h). (**H**) The tooth root is almost back into its original position (3 h + 9 h). (**I**) PDL bundles are stretched, the number of cells increased, cells remain haphazardly arranged. Alveolar bone surface shows deep staining with hematoxylin (3 h + 9 h). (**J**) There are many round cells in round types, there is a various array PDL fibroblasts (3 h + 3 d). (**K**) Tooth root movement is not seen (3 h + 3 d). (**L**) Many cells are oval in shape, the PDL fibroblasts are arranged on the root surface (3 h + 3 d). (**M**) PDL fibroblasts varies in shape, many cells are also uniquely oral in shape (3 h + 1 w). (**N**) Tooth root movement is not seen (3 h + 1 w). (**O**) PDL fibroblasts are mostly oval in shape, the PDL fibroblasts are arranged neatly on the root surface (3 h + 1 w). Copyright, 2012, Journal of Hard Tissue Biology.

## Data Availability

Not applicable.

## Appendix A

The Waldo method [19] was used to insert a separator between the right first and second maxillary molars to provide continuous mechanical load to the maxillary molar periodontium over a period of time. This separator comprised a rubber dam sheet (heavy force; Ivory, Premium rubber Dam Pure Latex; Heraeus Kulzer GmbH & Co. KG; Hanau, Germany) cut to approximately 2.0 mm × 2.0 mm and folded in half. The maxilla has three molars: first molar (M1), second molar (M2), and third molar (M3) from the mesial side. In addition, M1 and M2 have three roots, and M3 has two to three roots [4].

## Appendix B

Histopathological results by HE staining. Center column shows histopathological image of the alveolar bone socket morphology. Left column is high-power view of pressure side and right column tension side [26].
